# Culture-specific transcriptional drifts limit the fidelity of organoid infection models

**DOI:** 10.1371/journal.ppat.1014321

**Published:** 2026-06-04

**Authors:** Paula E. Schweizer, Wisal A. Elmagzoub, Sanaa M. Idris, Kamal H. Eltom, Julius B. Okuni, Lonzy Ojok, ElSagad Eltayeb, Ahmad Amanzada, Marianne Quaas, Gabriela Aust, Maxi Harzer, Thomas W. Vahlenkamp, Nico Jehmlich, Marlon R. Schneider, Guntram A. Grassl, Jens Puschhof, Knut Krohn, Ralph Goethe, Uwe Truyen, Jörg Galle, Ahmed Abd El Wahed

**Affiliations:** 1 Institute of Animal Hygiene and Veterinary Public Health, Leipzig University, Leipzig, Germany; 2 Department of Animal Health and Safety of Animal Products, University of Khartoum, Khartoum, Sudan; 3 Department of Pathology, Faculty of Veterinary Medicine, University of Khartoum, Khartoum, Sudan; 4 College of Veterinary Medicine, Animal Resources and Biosecurity, Makerere University, Kampala, Uganda; 5 Department of Pathology, Faculty of Medicine, Gulu University, Gulu, Uganda; 6 Faculty of Medicine, Al Neelain University/ Ibn Sina Specialised Hospital, Khartoum, Sudan; 7 Department of Gastroenterology and Gastrointestinal Oncology, Georg-August University Goettingen, Goettingen, Germany; 8 Research Laboratories and Clinic of Visceral, Transplantation, Thoracic, and Vascular Surgery, Leipzig University and University Hospital, Leipzig, Germany; 9 Institute of Virology, Faculty of Veterinary Medicine, Leipzig University, Leipzig, Germany; 10 Department of Molecular Toxicology, Helmholtz-Centre for Environmental Research GmbH, Leipzig, Germany; 11 Institute of Veterinary Physiology, Leipzig University, Leipzig, Germany; 12 Institute of Medical Microbiology and Hospital Epidemiology, Hannover Medical School, Hanover, Germany; 13 German Center for Infection Research (DZIF), Partner-Site Hannover-Braunschweig, Hanover, Germany; 14 Microbiome and Cancer Division, German Cancer Research Center (DKFZ), Heidelberg, Germany; 15 Interdisciplinary Center for Clinical Research, Faculty of Medicine, Leipzig University, Leipzig, Germany; 16 Institute for Microbiology, University of Veterinary Medicine, Hanover, Germany; 17 Interdisciplinary Centre for Bioinformatics (IZBI), Leipzig University, Leipzig, Germany; INEM: Institut Necker-Enfants Malades, FRANCE

## Abstract

Intestinal organoids are powerful tools for modeling host-pathogen interactions, yet culture-induced artifacts remain poorly defined. Here, we performed time-course transcriptomic profiling of patient-derived ileal organoids microinjected with *Mycobacterium avium* subsp. *paratuberculosis* (MAP) or treated with the swelling agent forskolin (FSK) and compared expression changes to untreated controls. Our analysis revealed that culture-associated transcriptional drift was the primary driver of gene expression changes over the 48-hour time course, likely obscuring pathogen-specific responses. This drift involved progressive downregulation of metabolic genes accompanied by the upregulation of genes with high GC content. Although FSK treatment improved. microinjection efficiency, it also introduced additional confounding effects, including transient activation of apoptotic pathways and upregulation of inflammatory genes that resolved within two days. While MAP infection did not produce significant single-gene responses, gene set analysis revealed significant infection-associated effects. Spatial transcriptomics further demonstrated that multiple enterocyte gene sets became downregulated during culture, independent of their normal crypt-villus expression pattern. Gene sets typically expressed at the crypt-villus junction showed the greatest downregulation. Notably, MAP infection selectively counteracted these culture-induced changes, preserving gene expression patterns characteristic of the upper villus epithelium. These findings indicate that both metabolic stress and pharmacological interventions can substantially confound organoid-based infection models. More broadly, they underscore the importance of spatially resolved transcriptomics and appropriate temporal controls to distinguish genuine pathogen responses from culture-related artifacts. To our knowledge, no previous studies have used human intestinal organoids to model MAP infection or characterized the epithelial transcriptomic response. This work establishes critical frameworks for investigating MAP’s role in Crohn’s disease pathogenesis.

## Author summary

Intestinal organoids - lab-grown mini-intestines - are increasingly used to study how infectious bacteria interact with human gut cells. However, our findings reveal unexpected challenges that may influence how infection studies are interpreted. We examined gene activity in intestinal organoids under four conditions: untreated, injected with a neutral solution, treated with forskolin (a chemical that could simplify infection processes), and infected with *Mycobacterium avium* subsp. *paratuberculosis* (MAP), a gut pathogen linked to Crohn’s disease.

We discovered that organoids naturally deteriorate during culture in a predictable pattern. Over 48 hours, key metabolic functions shut down, and normal cellular regulatory pathways become disrupted. These time-dependent changes were so strong that they likely masked most MAP-specific responses. Nevertheless, by applying advanced analysis techniques and spatial transcriptomics, we found that MAP infection was able to prevent the normal breakdown pattern and maintain gene activity patterns in different intestinal areas. We also observed that forskolin triggered stress responses and cell death pathways that can confound the interpretation of infection results.

Together, these findings indicate the need for caution when interpreting results of organoid infection experiments, emphasizing the importance of proper controls and analytics that distinguish genuine infection responses from culture artifacts.

## Introduction

Intestinal organoids have revolutionized our ability to model intestinal biology and disease. These self-organizing tissue cultures derived from stem cells faithfully mirror three-dimensional architecture, cellular diversity, and functional properties of the native intestinal epithelium [1,2]. Intestinal organoids serve as valuable tools for studying intestinal tissue development, disease modeling, drug discovery, and regenerative medicine applications [3–7].

Traditional models exhibit inherent limitations for studying the interactions between intestinal tissue and infectious microorganisms [8]. Several methodological approaches have been developed to introduce pathogens into organoids, including: 1) apical-out cultures exposing the luminal surface for direct pathogen contact; 2) microinjection delivering pathogens directly into the organoid’s hollow center; and 3) two-dimensional monolayer cultures on permeable membrane systems [9–12]. Among these, microinjection best preserves native three-dimensional architecture and an enclosed luminal compartment, which is thought to recapitulate the hypoxic conditions of the intestinal lumen [13,14]. Although this technique requires specialized equipment and technical expertise [14,15], we selected it as our infection approach to maintain physiological relevance (Fig 2).

*Mycobacterium avium* subsp. *paratuberculosis* (MAP) causes Johne’s disease in ruminants with significant economic impact and has been implicated as a potential cofactor in Crohn’s disease pathogenesis, though this remains controversial [16]. While organoid-based MAP studies remain largely confined to veterinary applications [17–20], the suitability of human intestinal organoid systems for modeling MAP infection remains underexplored.

A key technical consideration in our experimental design involves the use of forskolin (FSK). In organoid research, FSK is commonly used to assess epithelial function through the Forskolin-Induced Swelling Assay [21]. FSK activation of cAMP triggers chloride channel opening, particularly CFTR (Cystic Fibrosis Transmembrane Conductance Regulator) channels, creating an osmotic gradient that drives water influx and measurable organoid swelling [22,23]. This assay is well-established for studying cystic fibrosis pathophysiology and therapeutic interventions [24]. However, its application in infection models lacks published precedent, and the resulting physiological perturbations may confound infection-specific transcriptional responses. Nonetheless, transient lumen expansion may represent a promising approach to facilitate microinjection, particularly in morphologically irregular organoids (Fig 2).

RNA sequencing (RNA-seq) offers a powerful approach to characterize functional states and identify pathways governing organoid responses. Since culture variability can cause deviations from physiological conditions, RNA-seq is valuable for assessing organoid fidelity while uncovering gene expression patterns relevant to host–pathogen interactions and disease mechanisms [25–30].

To evaluate the performance of commercially available organoid culture systems for studying epithelial responses to intracellular enteric pathogens, we employ patient-derived human ileal organoids to investigate transcriptional dynamics following MAP infection. We evaluate the effects of FSK treatment as a potential facilitator of microinjection procedures. RNA-seq is employed to characterize transcriptional changes associated with organoid maturation, FSK treatment, and MAP infection. This approach provides important insights into host-pathogen interactions and illustrates the influence of culture-related conditions on gene expression profiles.

## Materials and methods

### Ethics Statement

Ethical approval for this research was obtained from the Ethics Committee of Leipzig University on November 2, 2021 (reference number: 2021.10.04_eb_118). All study procedures were conducted in accordance with institutional ethical guidelines and the Declaration of Helsinki. No human or animal samples were collected during this study. All organoids used in this research were commercially obtained from established suppliers.

Four experimental groups (n = 3–4 per group) were tested: control (untreated), PBS injection (vehicle control), forskolin (FSK) treatment (swelling agent), and MAP infection (target pathogen). Temporal sampling was conducted at 2h, 24h, and 48h post-treatment, with the MAP-infected group sampled only at 48h. Sample processing involved organoid harvest, RNA extraction, library preparation, and RNA sequencing. The bioinformatics analysis pipeline included primary analysis followed by three parallel approaches: Self-Organizing Map (SOM) analysis, pathway enrichment analysis, and spatial gene set analysis (crypt-villus axis). *Created in BioRender. Abd el wahed, A. (2026)* https://BioRender.com/320j4cp

### Study design

**Fig 1:**
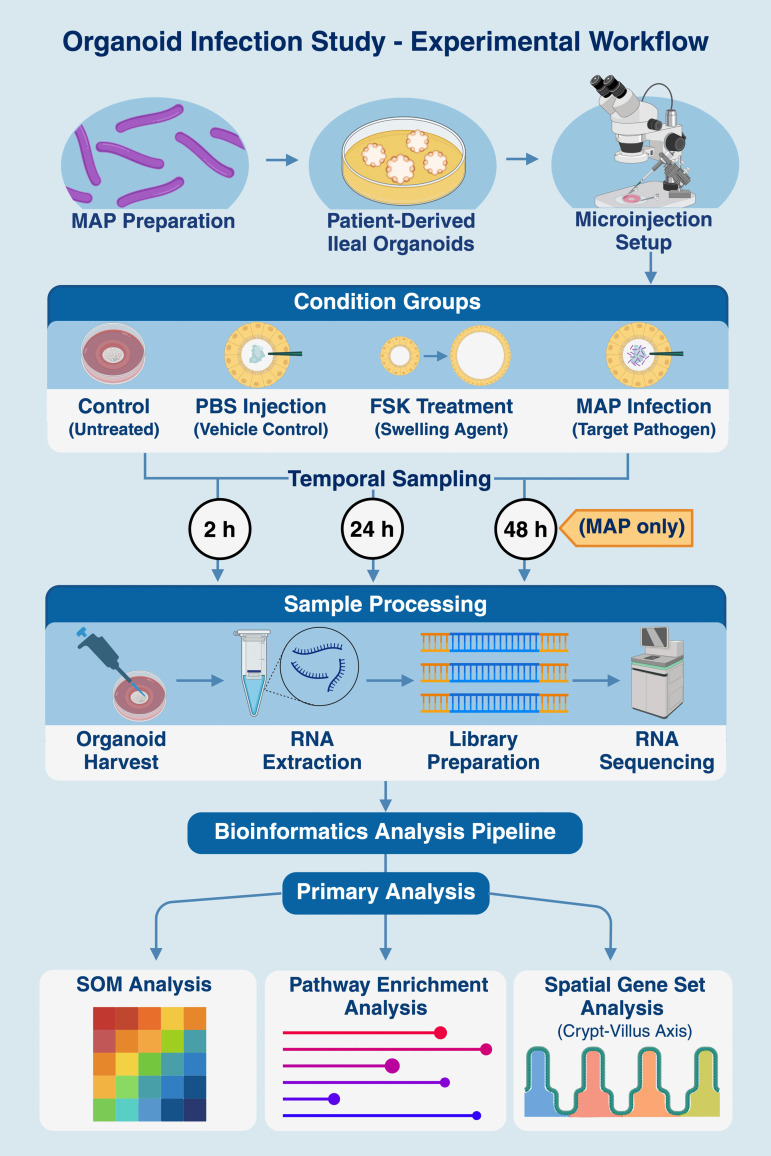
Experimental design workflow for patient-derived human ileal organoid infection study. This flowchart illustrates a multi-step protocol for studying intestinal organoids and their transcriptional responses to infection, treatment, and temporal progression. The study workflow begins with patient-derived ileal organoids that undergo culture establishment, *Mycobacterium avium* subsp. *paratuberculosis* (MAP) preparation, and microinjection setup.

This study employed commercially obtained patient-derived human ileal organoids to investigate transcriptional responses to MAP infection. Four experimental groups were compared: untreated controls (CON), PBS vehicle controls (PBS), forskolin-treated (FSK), and MAP-infected (MAP) (Fig 1). CON received no treatment; FSK had forskolin supplemented to the culture medium without injection. PBS and MAP underwent microinjection with PBS vehicle or MAP suspension, respectively; neither received FSK treatment. CON, PBS, and FSK were harvested at 2h, 24h, and 48h post-treatment, while MAP-infected organoids were harvested at 48h only. All organoids were subsequently processed for RNA extraction and sequencing library preparation as described below.

### Intestinal organoid culture and maintenance

Patient-derived human intestinal organoids were obtained from the Gastrointestinal Organoid Biobank (Sigma-Aldrich, cat. no. SCC339), derived from ileal tissue of a healthy 19-year-old male donor. Organoids were cultured in 24-well plates using 50 μL Matrigel domes with IntestiCult-SF Organoid Growth Medium (Human) (OGM) (Stemcell Technologies, Vancouver, Canada) and maintained at 37°C with 5% CO₂. For passaging, based on Lee et al. [31], Matrigel domes were resuspended in ice-cold DMEM/F12 and washed twice by centrifugation (300 × g, 5 min). Organoids were mechanically fragmented using BSA-coated pipette tips, then reseeded by mixing 25 μl of organoid suspension with 30 μl of fresh Matrigel (organoid:Matrigel ratio of 5:6). After 30 min polymerization at 37°C, 500 μL OGM was added. Organoids were passaged every 6–8 days with medium changes every 2–3 days. All experiments used organoids from the same donor on day 6 following passage 13, a passage number selected to ensure post-thaw stabilization and sufficient biomass expansion while remaining within the range of preserved cellular function [32,33].

### Microinjection into intestinal organoids

**Fig 2:**
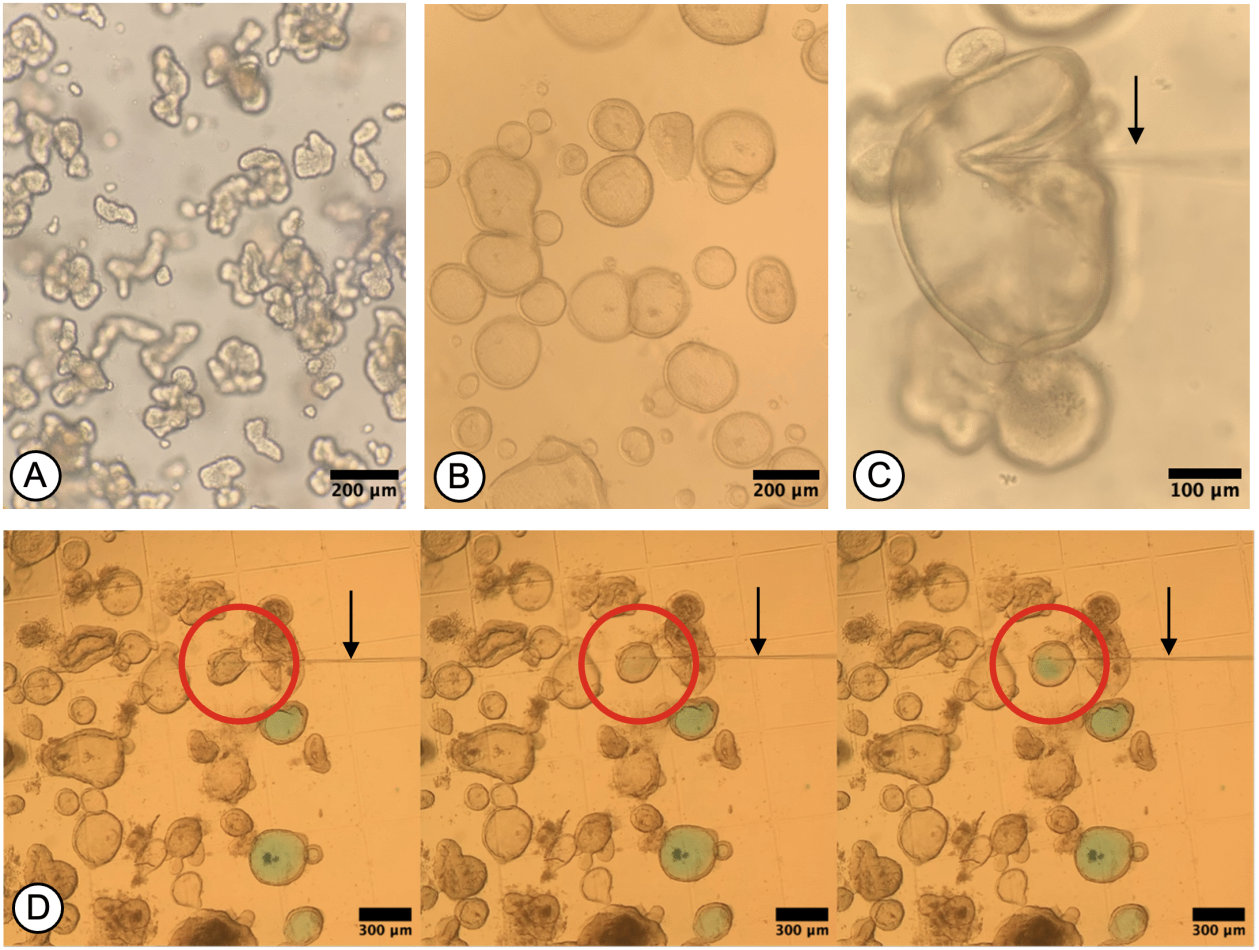
Technical challenges and methodology in organoid microinjection. **(A)** Budded intestinal organoids displaying complex 3D morphology with multiple protruding structures, which presents technical challenges for precise microinjection. **(B)** Cystic intestinal organoids present simplified spherical morphology that enables easier microinjection access, while illustrating the size heterogeneity characteristic of organoid cultures. This morphology was aspired for this study. **(C)** Pre-injection position of glass microcapillary (black arrow) adjacent to intestinal organoid, demonstrating the flexible nature of the epithelial membrane prior to penetration during microinjection procedure. **(D)** Sequential microinjection process showing progressive delivery of injection solution containing 0.05% Fast Green Dye into the organoid lumen. The time-course images demonstrate increasing dye intensity and corresponding organoid volume expansion, confirming successful intraluminal delivery and microcapillary (black arrow) positioning within the organoid cavity. **Note that the injection volume applied was intentionally excessive for clear visualization purposes in this demonstration.**

For microinjection, 30 organoids were cultured in 20 μL Matrigel domes (70% concentration) in 35 mm μ-dishes (Ibidi, cat. no. 80156) for 6 days until they reached sufficient diameter for microinjection, following the protocol of Puschhof et al. [13]. MAP (DSM 44133) was obtained from DSMZ (Braunschweig, Germany) and cultured in Middlebrook 7H9 broth with mycobactin J, Tween 80, and OADC at 37°C for 14 days. The multiplicity of infection (MOI) was calculated at 1:30 based on ~1,300 cells per organoid. Bacterial concentration was determined using McFarland densitometry (Grant Instruments, Cambridge, UK) calibrated to F57 qPCR counts based on Slana et al. [34]. Microinjections used a FemtoJet (Eppendorf) injector with custom 6 μm microcapillaries (35° bent) (Biomedical Instruments, Zöllnitz, Germany) (Fig 2). Fast Green Dye (0.05%) enabled visual confirmation. Injection parameters: 350 hPa for 1–3 s (dependent on organoid size), delivering 10–30 nL volumes. F57 qPCR confirmed absence of cross-contamination post-injection.

### Generation of FSK-treated intestinal organoids

#### Intestinal organoids were prepared using identical methodology as microinjection experiments.

At time point 0, FSK was added to the organoid growth medium at 10 μM final concentration to induce swelling (Fig 7). The assay was performed under identical environmental conditions as microinjection experiments to ensure comparability.

### RNA extraction and DNase digestion

Intestinal organoids were harvested for RNA extraction at predetermined time points (control and FSK-group: 2 h, 24 h, and 48 h post-treatment; infected group: 48 h post-infection only). Total RNA was isolated using the RNeasy Mini Kit (Qiagen, Hilden, Germany) following the manufacturer’s protocol with minor modifications. Medium was aspirated and cells lysed with 700 μL Buffer RTL for 3 min on a rocking platform. Lysates were mixed with 700 μL of 70% ethanol and applied to RNeasy spin columns. Columns were washed with Buffer RW1, treated with DNase I incubation mix for 15 min at room temperature, then washed with remaining RW1 buffer and twice with Buffer RPE. RNA was eluted in 30 μL nuclease-free water and stored at -80 °C until analysis.

### RNA-seq

RNA-seq libraries were prepared from n = 3 technical replicates per condition and time point, except for MAP-infected organoids harvested at 48h (n = 4), yielding 31 samples in total. Strand-specific random primed library preparation was performed using 150 ng of total RNA with the Watchmaker RNA library prep kit with Polaris depletion (Watchmaker Genomics), following the manufacturer’s instructions. Paired-end sequencing (2 × 150 bp) was performed on an Illumina NovaSeq 6000 at the sequencing core facility of the Faculty of Medicine, University of Leipzig. After demultiplexing with bcl2fastq software (Illumina, v2.20) and quality trimming with FASTP [35], reads were mapped to the human reference genome (hg38) using HISAT2 [36]. StringTie and the R package Ballgown [37] were employed for transcript quantification. Raw counts were normalized using DESeq2 [38], which has been recommended for comparative RNA-seq analysis [39].

### Self-Organizing MAP (SOM) analysis

To systematically explore transcriptional patterns across multiple experimental conditions and time points without predetermined assumptions, we employed SOM implemented in the oposSOM pipeline [40]. This unsupervised machine learning approach reduces high-dimensional transcriptomic data into interpretable two-dimensional representations while preserving topological relationships, enabling identification of co-expressed gene sets. DESeq2-normalized RNA-seq data were clustered using a 30 × 30 metagene grid with default parameterization [40], with each metagene representing genes sharing similar expression patterns.

### Gene set enrichment and GC content analysis

The analysis was performed using the gene set enrichment analysis tool ShinyGO 0.82 [41].

Charts are taken from this pipeline.

### Statistics

Data and image analyses were conducted using statistics software R [42]. We adjusted significance tests for multiple comparisons using the Benjamini-Hochberg method, identifying significant associations at 5% FDR. Differential expression analysis of gene sets was performed applying CAMERA [43] using the R-package limma.

## Results

### Transcriptomic profiling of organoid infection models across multiple conditions and time points

Organoid-based infection models offer valuable insights into host-pathogen interactions, but technical artifacts from experimental protocols can confound biological interpretations. To address this challenge and optimize infection protocols, patient-derived human ileal organoids were analyzed using RNA-seq to distinguish genuine infection responses from experimental artifacts. The study examined four conditions: untreated controls, PBS controls, FSK-treated organoids, and MAP-infected organoids across multiple time points (2h, 24h, 48h posttreatment; 48h post-infection). After quality control, 31 samples underwent differential gene expression analysis.

### SOM analysis reveals temporal changes in gene expression

**Fig 3:**
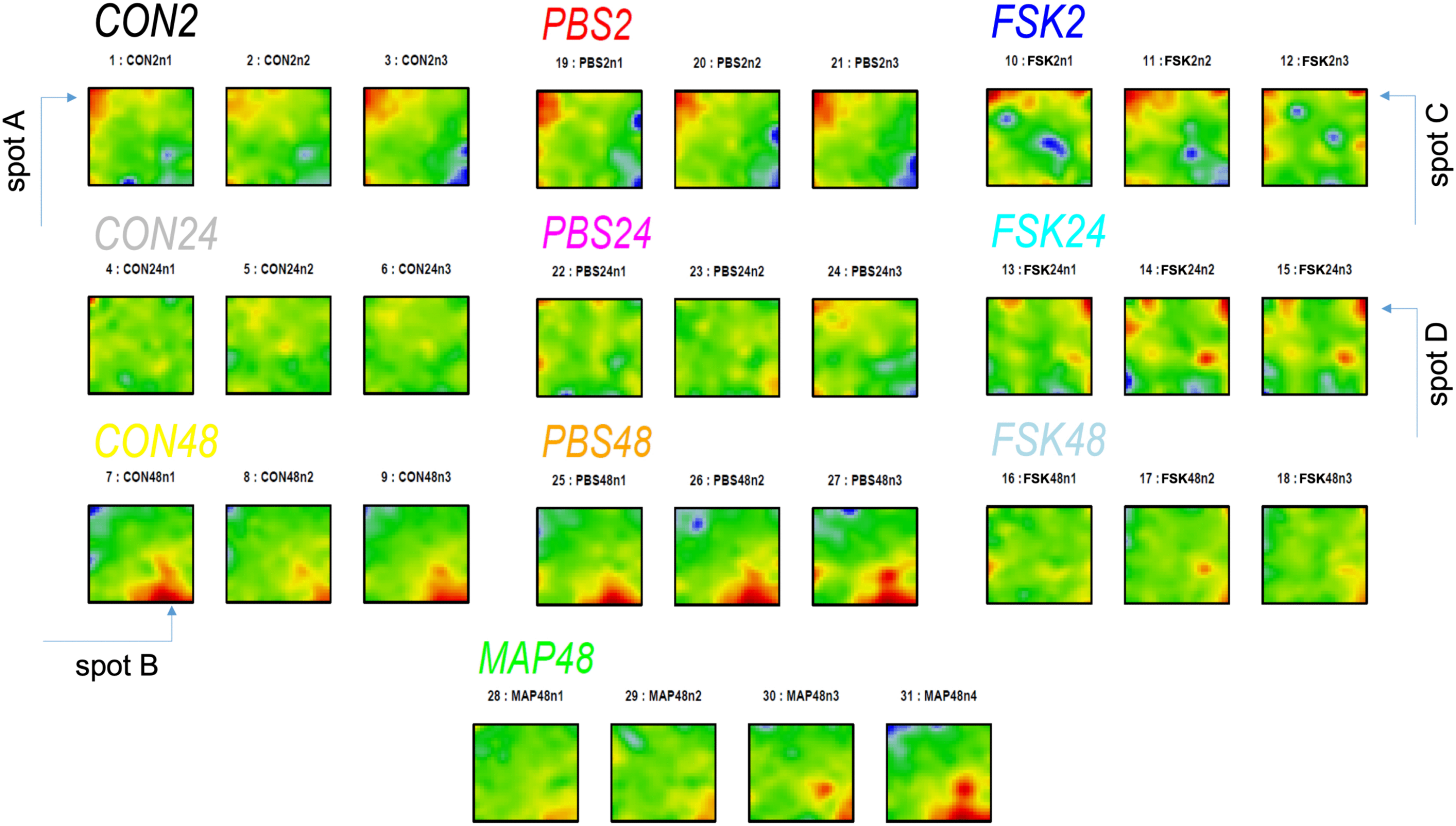
Visualizations of gene regulatory dynamics by SOM analysis. SOM portraits display metagene expression patterns at three time points (2h, 24h, 48h) across four experimental conditions: untreated controls (CON), PBS vehicle controls (PBS), forskolin-treated organoids (FSK), and MAP-infected organoids (MAP; 48h only). An absolute color scale was applied uniformly across all samples. Red metagenes (MGs) indicate transcriptional activation; blue MGs indicate downregulation. MGs activated at early time points (2 h, spot A) become downregulated over time (24 h, 48 **h)**. MGs repressed at early time points (2 h, spot B) become activated over time (24 h, 48 **h)**. FSK-samples show two additional spots specifically activated at 2 h (spot C) and 24 h (spot **D)**. n = 3 technical replicates per group except MAP48h (n = 4).

Self-Organizing Map (SOM) clustering analysis was conducted to explore sample relationships and ensure data integrity across intestinal organoids transcriptomes (Fig 3 and S1 Fig). This unsupervised machine learning approach transforms high-dimensional gene expression data into two-dimensional topological maps. The analysis assigns genes with similar expression patterns across all samples to so-called metagenes (MGs), the building units of the SOM. Expression of these MGs within an individual sample defines the SOM portrait of that sample. Throughout the SOM, neighboring MGs show similar expression, thus potentially form over- and under-expression spots within a portrait. Following SOM-analysis, unsupervised clustering is applied to identify clusters of MGs that form these spots.

Our analysis revealed consistent temporal expression changes across experimental groups (CON, PBS, FSK), with the same pattern observed in an independent 48 h group (MAP). We define this phenomenon as transcriptional drift, characterized by monotonous changes in gene expression over time, mathematically expressed as ∆E/∆t ≠ 0, where E represents gene expression level and t denotes experimental time points. In the SOM portraits from the early experimental phase (2 h), upregulated MG clusters localized to the upper left corner

(red, spot A), while downregulated clusters localized to the lower right corner (blue, spot B). Over time, expression levels of spots A and B inverted. MAP-infected samples at 48 h closely resembled other groups at this timepoint, indicating limited MAP-induced expression changes.

Complementary, pairwise correlation analysis of MG data corroborated these temporal dynamics (S2 Fig). Most 2 h samples across conditions showed strong positive correlations, indicating synchronized early responses (all CON and PBS replicates, one FSK replicate; n = 7). Some 24 h samples maintained positive correlations with 2 h samples (two CON replicates, one PBS replicate), suggesting persistent early transcriptional programs. At 48 h, samples exhibited negative correlations with earlier timepoints but strong positive correlations among themselves, confirming a fundamentally different transcriptional state.

These temporal clustering patterns indicate significant transcriptional changes throughout the experimental timeline. This intrinsic temporal drift (∆E/∆t ≠ 0) may have confounded detection of treatment-specific responses, particularly at later timepoints where system-wide reorganization predominated over treatment-induced effects.

### Pathway analysis reveals distinct functional and epigenetic programs

SOM analysis revealed two distinct clusters (spot A and B), each associated with specific gene sets (A and B), with opposing temporal dynamics. To gain insight into the biological processes underlying this clustering, we analyzed each gene set in more detail.

**Fig 4:**
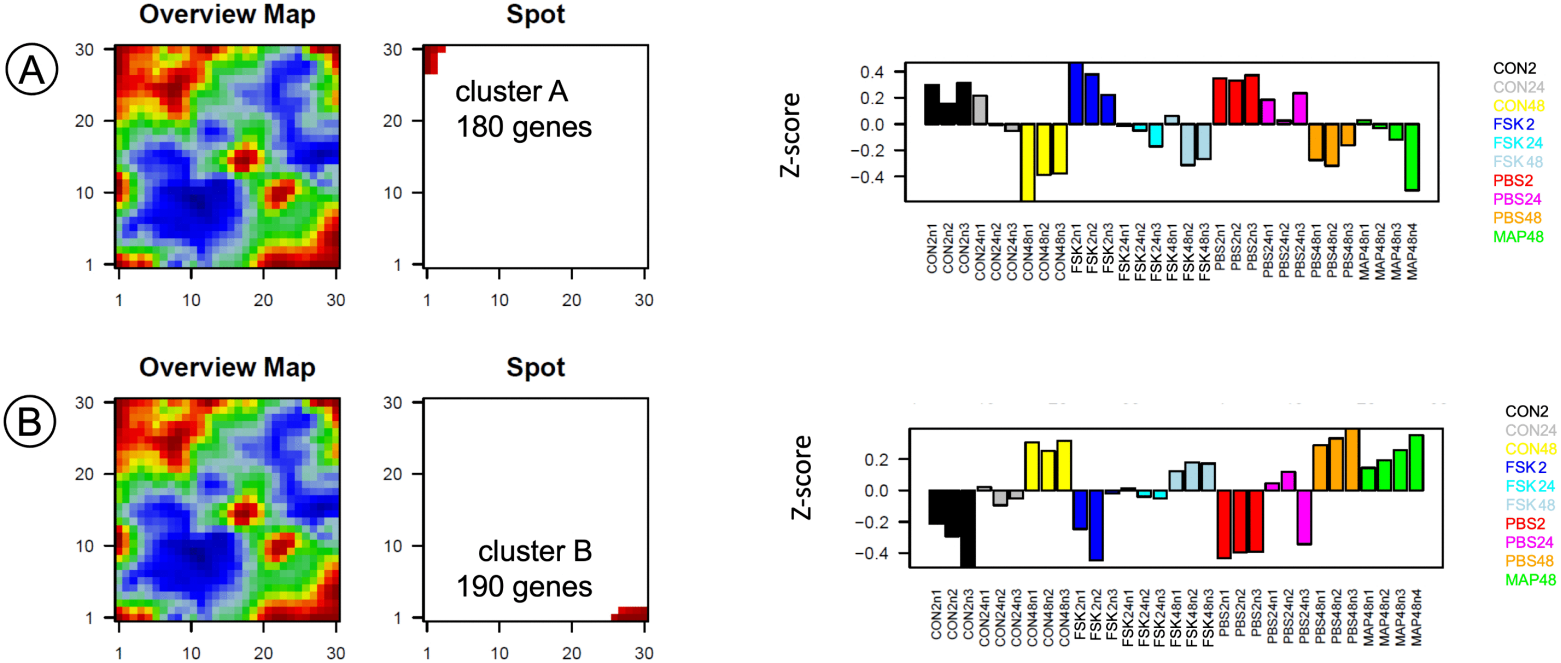
Consistent temporal dynamics of gene expression across all experimental groups. The overview maps (left) display the spatial organization of gene expression patterns from low (blue) to high (red), identified by SOM analysis. Middle panels highlight the selected cluster for detailed analysis. Right panels display expression Z-scores for genes within each cluster across experimental groups (CON2-MAP48), with colored bars representing different conditions as indicated in the legend. n = 3 technical replicates per group except MAP48 (n = 4); MAP-infected organoids were profiled at 48h only. **(A)** Progressive downregulation: genes exhibit high expression at 2 h, followed by gradual decreases at 24 h and 48 h across all groups. **(B)** Progressive upregulation: genes exhibit low expression at 2 h, followed by gradual increases at 24 and 48 h across all groups.

Gene set A (180 genes, ∆E/∆t < 0) is activated at the 2 h time point across all conditions (CON, PBS, FSK), followed by progressive downregulation at 24 and 48 h (Fig 4A). MAP-infected samples at 48 h matched with the uninfected groups at that time point.

**Fig 5:**
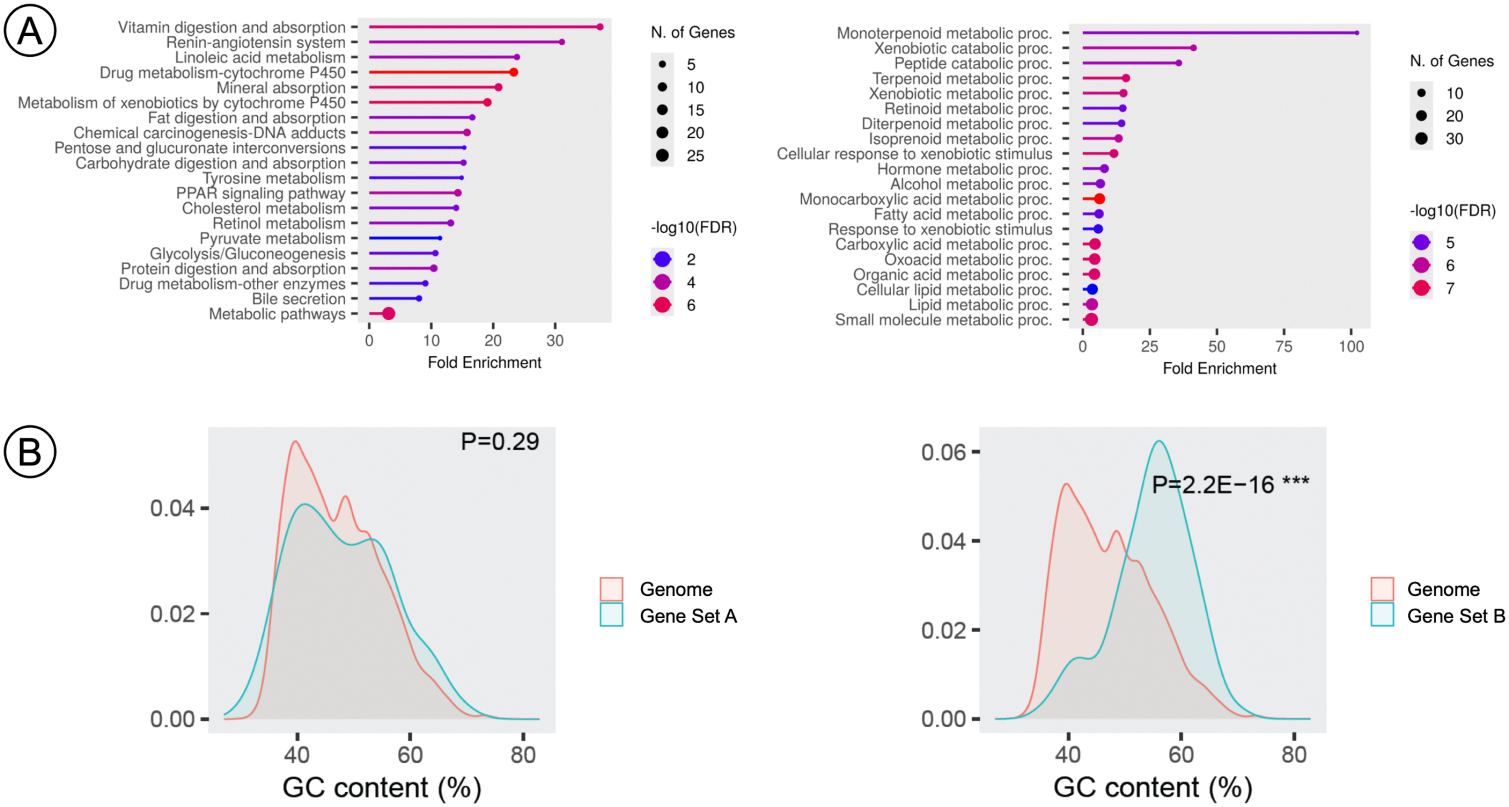
Biological processes behind expression dynamics linked to metabolism and epigenetics. **(A)** KEGG (left) and GO (right) enrichment analysis for gene set A, performed using ShinyGO 0.82. Fold enrichment values are displayed on the x-axis; circle size indicates the number of genes per pathway and circle color indicates statistical significance. Significant enrichment (FDR < 0.05, Benjamini-Hochberg correction) was observed for key metabolic processes, including fat, carbohydrate, protein, vitamin, and mineral metabolism, as well as drug metabolism pathways. genes in each pathway and circle color representing statistical significance. The predominant enrichment of digestive and absorptive pathways highlights the functional maturity of the organoid model in recapitulating intestinal metabolic functions. **(B)** GC content distribution for gene sets A (left) and B (right) relative to the genome-wide distribution. Genes in set A show the expected distribution, whereas genes in set B display substantially elevated GC content.

KEGG pathway analysis revealed enrichment of nutrient processing capabilities during early phases, including carbohydrate, lipid, protein, vitamin, and mineral digestion pathways (Fig 5A). Bile secretion pathway enrichment indicated ileal-specific differentiation, while xenobiotic metabolism pathways suggested functional barrier properties. GO analysis confirmed declining metabolic specialization including terpenoid metabolism, organic acid processing, and integrated stress response systems.

These findings demonstrate that organoids possessed mature intestinal metabolic capacity early, which progressively decreased over time.

Gene set B (189 genes, ∆E/∆t > 0) displayed the inverse expression pattern to set A, with increased expression over the same time course (Fig 4B). Unlike set A, these genes lacked significant

KEGG or GO enrichment but displayed elevated GC content relative to genome average (Fig 5B). The combination of high GC content and lack of functional enrichment raises the possibility that gene activation within set B is associated with a characteristic sequence enabling activation.

Collectively, these results suggest temporal expression changes reflect both functional metabolic programs (set A) and sequence-specific, likely chromatin remodeling events (set B) during organoid culture.

### FSK treatment effects on intestinal organoid gene expression

**Fig 6:**
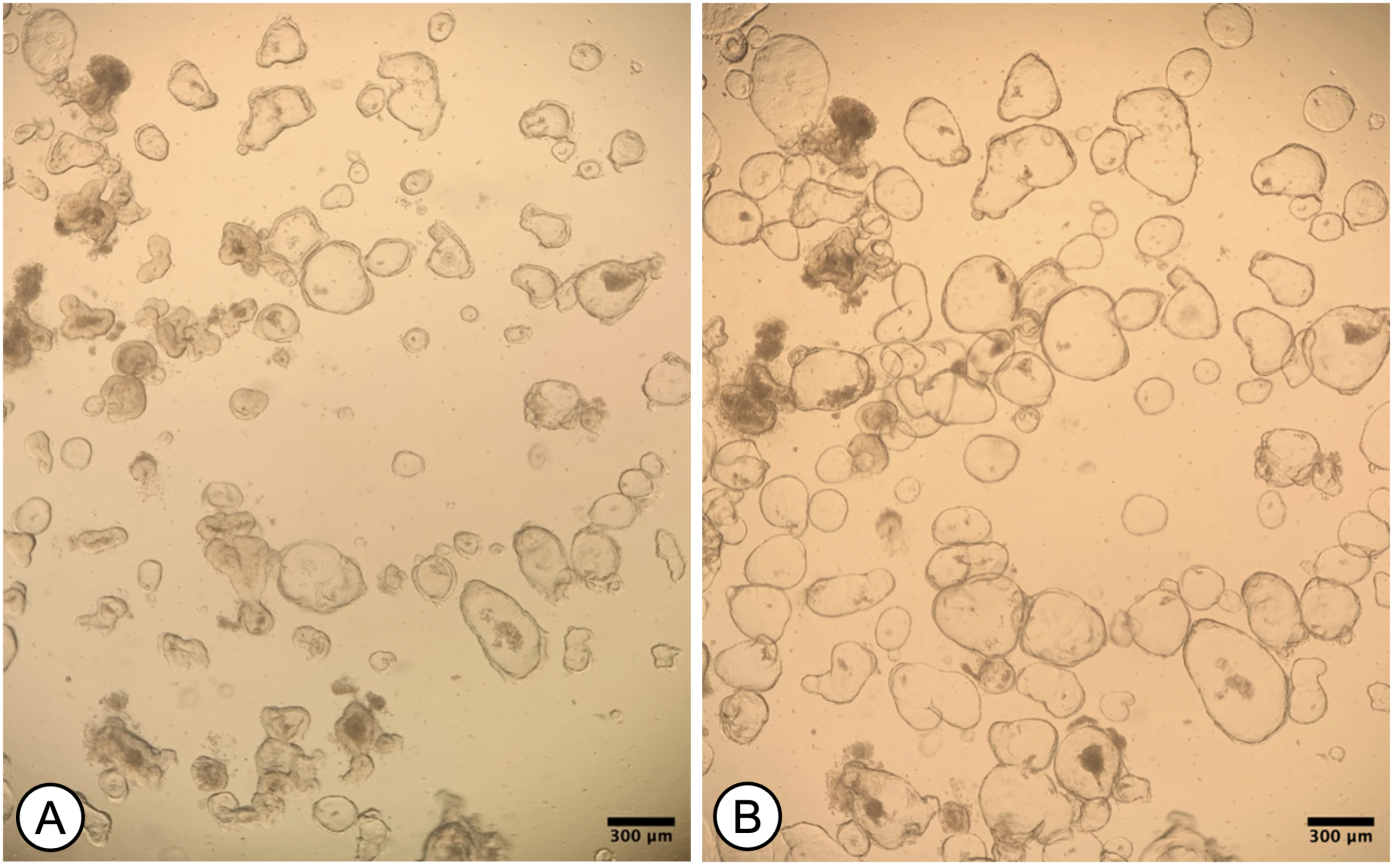
Morphological transformation of human intestinal organoids following forskolin treatment. **(A)** Before forskolin (FSK) treatment, intestinal organoids display typical spherical to budded morphology with well-defined epithelial architecture. **(B)** Following 1 h FSK treatment, organoids exhibit pronounced cyst-like swelling and epithelial thinning resulting from cAMP-mediated fluid secretion into the lumen.

FSK-induced swelling facilitated microinjection into organoids with variable shapes and sizes (Figs 2 and 6). However, as a cAMP activator, FSK modulates multiple cellular pathways, requiring assessment of its impact on gene expression.

**Fig 7:**
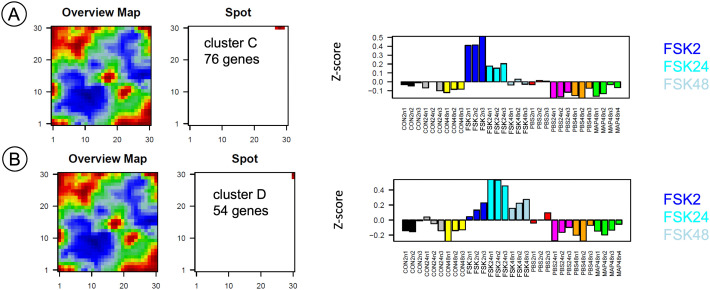
Detection of forskolin-specific gene expression dynamics. The overview maps (left) display the spatial organization of gene expression patterns from low (blue) to high (red), identified by SOM analysis. Middle panels highlight the selected cluster for detailed analysis. Right panels display expression Z-scores for genes within each cluster across experimental groups (CON2-MAP48), with colored bars representing different conditions as indicated in the legend. n = 3 technical replicates per group except MAP48h (n = 4); MAP-infected organoids were profiled at 48h only. Two clusters (C and D) with contrasting temporal responses to forskolin (FSK) were identified: **(A)** Cluster C shows early activation at 2 h, whereas **(B)** Cluster D exhibits delayed activation at 24 **h.** Both clusters return to baseline by 48 h, indicating temporally regulated transcriptional programs.

**Fig 8:**
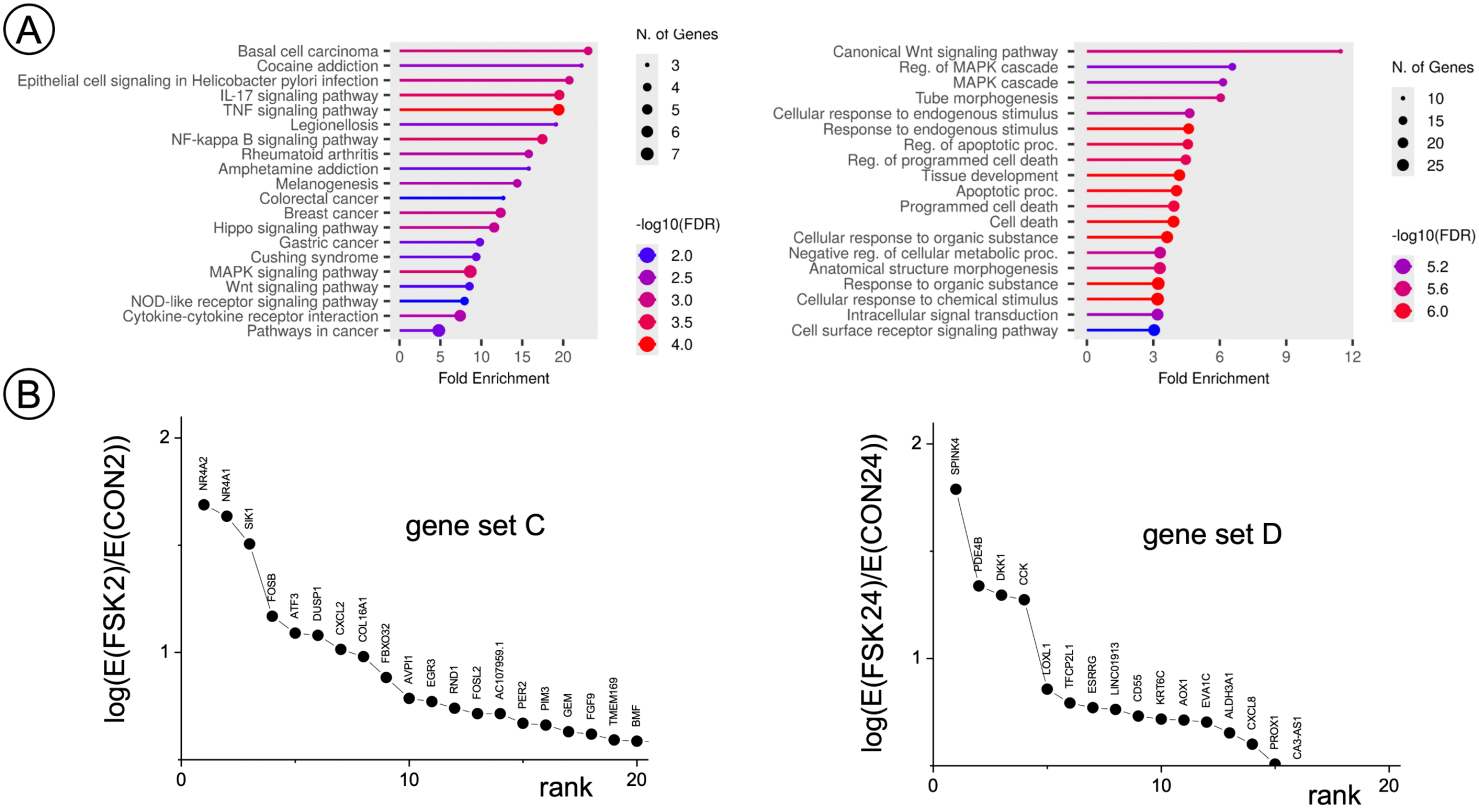
Functional annotation of gene sets associated with forskolin-specific clusters. **(A)** Enrichment analysis showing KEGG pathways (right) and GO biological processes (left) overrepresented in gene set C, performed using ShinyGO 0.82. Fold enrichment values are displayed on the x-axis, with circle size indicating the number of genes in each pathway and circle color indicates statistical significance (FDR < 0.05, Benjamini-Hochberg correction). Enriched pathways include canonical Wnt signaling, MAPK cascade regulation, apoptotic processes, programmed cell death, and cellular responses to various stimuli, suggesting coordinated activation of cell death mechanisms and stress response pathways. **(B)** Expression fold-changes for genes in set C (left) and set D (right) following forskolin treatment relative to untreated controls at the corresponding time point. All results are significant at FDR < 0.05 (Benjamini-Hochberg correction). n = 3 technical replicates per group.

SOM analysis identified two distinct temporal clusters: gene set C showed rapid upregulation within 2 h, while gene set D peaked at 24 h, with both returning to baseline by 48 h (Fig 7). GO and KEGG pathway enrichment analysis of the rapid-responding gene set C revealed significant enrichment in cell death and apoptotic processes, with 5 of the top 20 GO terms directly related to apoptosis (GO:0042981, GO:0043067, GO:0006915, GO:0012501, GO:0008219) (Fig 8A). Additionally, immune-related pathways were upregulated, including TNF signaling (hsa04668), NF-κB signaling (hsa04064), and cytokine-cytokine receptor interaction (hsa04060). Gene set D showed no significant pathway enrichment.

Differential expression analysis confirmed upregulation of cAMP signaling components, including nuclear receptors (NR4A1, NR4A2), AP-1 transcription factors (FOSB, FOSL2), and negative feedback regulators (SIK1, DUSP1, PDE4B) (Fig 8B).

These findings demonstrate that FSK treatment induces cellular stress responses extending beyond swelling effects, potentially confounding infection-related gene expression analysis.

### Distinct tissue compartments undergo specific regulation

Intestinal organoids recapitulate the structure and cellular composition of *in vivo* tissue. We investigated whether temporal and condition-dependent changes in gene expression correspond to alterations in specific tissue compartments.

Using spatial expression data from Harnik et al. [44] covering crypts and six villus zones (V1-

V6, lower to upper villus), SOM analysis identified “bottom set” genes (cluster EM) with highest crypt expression and “tip set” genes (cluster DM) upregulated in upper villus zones (S3A, S3B Fig). Note that this analysis assesses changes in the activation of zone-specific differentiation programs rather than the spatial distribution of cell types within organoids. The latter would require spatial transcriptomic approaches. Both sets are significantly downregulated in control samples at 48 h (CON48) compared to 2 h (CON2), though no individual gene of the sets reached significance (p < 0.05) after correction for multiple testing. The bottom set exhibited more pronounced regulation than the tip set, indicating preferential suppression of crypt and lower villus genes over time, whereas genes expressed in upper regions may be upregulated (S3C Fig). Consistent with this pattern, eight mitochondrial genes in the tip cluster (*MT*-*ND1*, -*ND2*, -*ND3*, -*CO1*, -*CO2*, -*CO3*, -*ATP6*, -*CYB*) encoding oxidative phosphorylation (OXPHOS) complex subunits were upregulated over time, suggesting high metabolic activity of mature villus-tip enterocytes.

**Fig 9:**
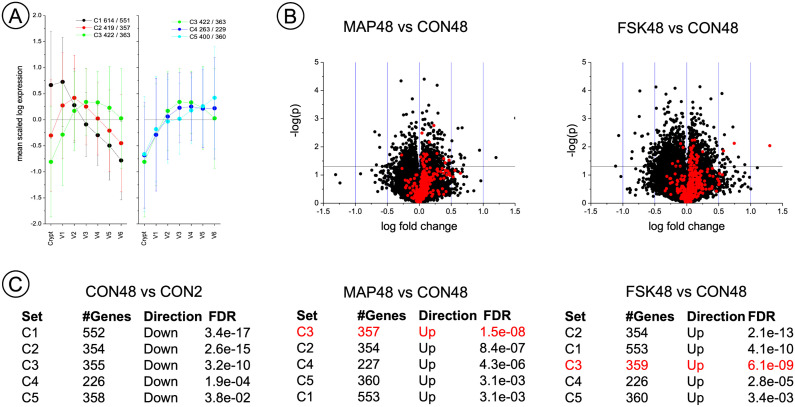
Regulation of zonated gene sets along the crypt-villus axis. **(A)** Expression profiles of gene sets from Moor et al. [45] along the crypt-villus axis. For each set, the number of mouse genes and expressed human orthologous genes are indicated. Expression values are scaled (sum across zones = 0 for each gene). Error bars show standard deviations, reflecting variability among genes within each set. Expression peaks in mouse villus regions C1–C5 correspond to human villus regions V1–V4 and V6, respectively, demonstrating conserved spatial gene expression patterns between species. **(B)** Volcano plots showing differential expression of individual genes comparing MAP-infected vs. control samples (MAP48 vs. CON48, left) and forskolin-treated vs. control samples (FSK48 vs. CON48, right) at the 48 h time point. All genes shown in black; genes from set C3 highlighted in red, indicating activation of this gene set. P-values are unadjusted for multiple testing and are shown for exploratory visualization purposes only. n = 3 (CON48, FSK48); n = 4 (MAP48). **(C)** Differential expression of gene sets C1 – C5 across three comparisons: control samples at 48 h vs. 2 h (CON48 vs. CON2, left), MAP-infected vs. control samples at 48 h (MAP48 vs. CON48, middle), and forskolin-treated vs. control samples at 48 h (FSK48 vs. CON48, right), assessed using CAMERA (limma). Both MAP infection and forskolin treatment induced upregulation across all crypt-villus zones relative to controls. FDR values were calculated using the Benjamini-Hochberg method; significant associations were identified at FDR < 0.05. n = 3 per group except MAP48 (n = 4). Gene numbers may vary due to missing expression values in individual samples.

Validation of the results was performed using five spatially-distinct enterocyte gene sets (C1 - C5, crypt-proximal to villus-tip) from mouse villi described by Moor et al. [45]. Orthologous human genes (>85% conservation) displayed similar zonation patterns, with expression maxima in human villus regions V1 - V4 and V6, respectively (Fig 9A). All sets were downregulated in control samples at 48 h (CON48) versus 2 h (CON2), with decreasing statistical significance in progressively higher zones (C1 > C2 > C3 > C4 > C5; Fig 9C). A similar but attenuated effect is observed when comparing 24 h (CON24) with 2 h (CON2) samples. PBS-injected samples exhibited identical dynamics, reinforcing preferential downregulation of lower villus genes.

Together, progressive downregulation of crypt-proximal genes (C1 - C3) combined with maintenance or upregulation of villus-tip genes (C4 - C5) and mitochondrial OXPHOS genes suggests a temporal transcriptional shift toward a more differentiated, villus-tip-like state during 48-hour culture.

These changes potentially mask infection-specific alterations. To enable comparison of FSK treatment effects with MAP infection responses, both conditions were analyzed against the non-treated control group (CON; comparison of MAP48 vs PBS48 is provided in S5 Fig). No genes showed significant differential expression between MAP48 and CON48 samples after adjusting for multiple testing (Fig 9B). However, gene set analysis revealed that MAP infection upregulated the tip set and all enterocyte sets compared to controls, indicating MAP modulates metabolic state and suppresses temporal drift (S3C Fig; Fig 9C). The effect was most pronounced in set C3, suggesting preferential infection of upper villus differentiated cells.

Beyond the spatial gene sets, we identified individual genes showing the same expression dynamics - decreased expression in controls but maintained expression in MAP-infected cells. Sedoheptulokinase (SHPK or CARKL) exhibited the strongest MAP-specific regulation and was also preserved under FSK treatment, though this effect did not reach significance after multiple testing correction (S4 Fig, p < 0.001). FSK treatment further elevated enterocyte-associated gene sets relative to controls, with pronounced upregulation in lower villus regions (C1-C2; Fig 9C), indicating overlapping transcriptional effects between FSK treatment and MAP infection responses.

## Discussion

**Fig 10:**
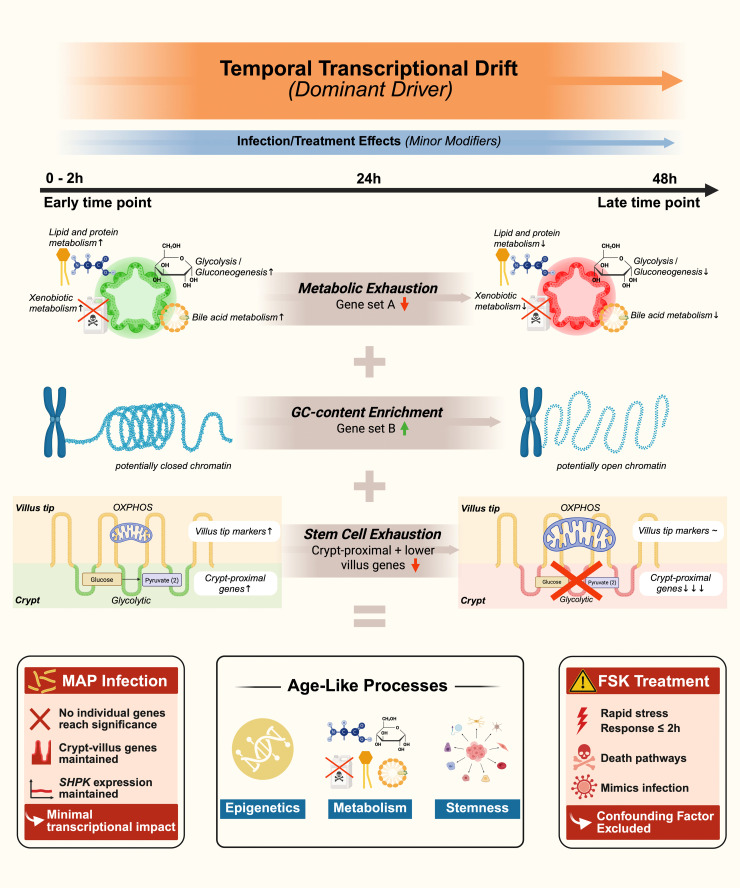
Schematic overview of transcriptional drift in patient-derived ileal organoids over the experimental timeline. This is a simplified schematic representation summarizing key findings discussed in this section; actual transcriptional changes are more complex and heterogeneous than depicted. The schematic serves as a conceptual framework and should not be interpreted as quantitatively precise or comprehensive of all transcriptional alterations observed. *Created in BioRender. Abd el wahed,* ***A.*** *(2026)* https://BioRender.com/xmlmu82.

Our transcriptomic analysis of patient-derived ileal organoids provides evidence that intrinsic temporal transcriptional drift potentially overshadows infection-specific responses. Importantly, these organoids had already undergone 6 days of culture following passage before the 48-hour experimental period, representing a total of 8 days in culture. Gene expression patterns were predominantly determined by culture duration rather than experimental condition, with all groups converging on similar transcriptional profiles at 48 h (Fig 10). This aligns with other studies showing that pathogen infection has minimal impact on transcriptional variance compared to biological factors like tissue origin and donor variability [46–48], raising fundamental questions about interpretability of infection studies at extended timepoints.

Consistent with this, human intestinal organoid infection studies commonly report modest transcriptional responses, encompassing 32–238 detected differentially expressed genes [49–51].

This temporal drift manifested as progressive loss of mature intestinal functions through downregulation of specialized metabolic pathways, evidenced by analysis of gene set A. At early timepoints, our organoids demonstrated sophisticated metabolic capacity encompassing all major nutrient classes, bile acid reabsorption, and xenobiotic metabolism - hallmarks of functionally mature ileal enterocytes [52–54]. However, this metabolic competence systematically declined over 48 h potentially due to metabolic exhaustion from limited nutrient availability, absence of luminal factors, or waste accumulation.

Concurrently, we observed progressive activation of gene set B, characterized by elevated GC content without functional pathway enrichment. One possible explanation for this pattern involves cellular adaptation through preferential activation of GC-rich promoters containing CpG islands susceptible to chromatin remodeling during stress [55,56]. If chromatin remodeling underlies these expression changes, nutrient limitation-induced chromatin reorganization could represent a contributing mechanism [57–62], though this remains to be further investigated.

The temporal downregulation of crypt-proximal and lower villus genes, combined with maintenance of villus-tip markers and upregulation of mitochondrial OXPHOS genes, may indicate a spatial reorganization of the intestinal epithelial architecture during culture. We hypothesize that declining glucose availability could preferentially support the survival of terminally differentiated villus-tip enterocytes, which are metabolically adapted for oxidative phosphorylation, while compromising the viability of glycolysis-dependent crypt stem cells. This interpretation is consistent with studies demonstrating that Lgr5 + stem cells exhibit characteristic glycolytic metabolism under glucose-rich conditions, whereas differentiated enterocytes display active oxidative phosphorylation [61]. Additional plausible mechanisms for stemness loss could include gradual depletion of growth factors (Wnt, R-spondin, and Noggin), changes in extracellular matrix composition and mechanical properties, and overcrowding effects that may develop during culture [63–66]. However, distinguishing the relative contributions of these factors and confirming spatial reorganization versus selective cell loss requires further validation.

While the underlying mechanisms remain unclear, these coordinated changes appear to mirror certain aspects of interconnected hallmarks of aging [67–69]: changes that may reflect stem cell exhaustion through progressive downregulation of crypt-proximal genes, possible epigenetic alterations via chromatin remodeling, and metabolic reprogramming characterized by OXPHOS upregulation coupled with glycolysis downregulation. However, the compressed timeline necessitates careful interpretation of whether suboptimal culture media conditions could potentially accelerate aging-like processes, as previously observed in brain organoids [70]. Further investigation is needed to distinguish culture-induced artifacts from genuine aging-related processes.

Various optimization methods show potential for reducing temporal drift effects. Perfusion-based “gut-on-a-chip” systems with continuous media exchange better maintain differentiated functions [71–73], while unified culture media that balance stem cell expansion with differentiation help preserve physiological gradients [74]. More frequent media changes could prevent metabolic exhaustion, and chromatin-stabilizing supplements like histone deacetylase inhibitors may enhance physiological relevance [75].

Against this backdrop of temporal drift, MAP infection showed minimal direct transcriptional response, with no individual genes reaching significance after multiple testing correction. This could reflect cellular stress masking pathogen-specific responses, inadequate infection efficiency, or successful immune evasion [76]. Interpreting this finding is complicated by the absence of prior studies examining MAP infection in human intestinal organoids or characterizing epithelial transcriptomic responses to this pathogen.

However, gene set analysis reveals that MAP infection counteracts temporal transcriptional drift observed in uninfected organoids, maintaining expression of crypt-villus genes that otherwise decline over 48 h. MAP appeared to preferentially modulate the C3 gene set (expressed in the upper villus), suggesting either preferential infection of cells with this differentiation profile or specific alteration of this transcriptional program. Notably, no prior studies have examined MAP’s preference for infecting specific spatially organized enterocytes along the villus axis. This counter-regulatory effect may reflect bacterial manipulation of host metabolism to maintain a permissive intracellular environment or a host response to maintain epithelial barrier function [77].

*SHPK* expression was maintained in MAP-infected organoids, which is notable given its role in pentose phosphate pathway regulation and NADPH-dependent antioxidant defense [78]. Though *SHPK* suppresses glycolysis in immune cells [78,79], its intestinal epithelial function is unknown. This maintenance may reflect bacterial metabolic manipulation or host NADPH-mediated defense. Despite lacking statistical significance after multiple testing correction, *SHPK* persistence in FKS-treated samples suggests a potentially important metabolic adaptation warranting investigation.

FSK treatment, while successfully facilitating microinjection through organoid swelling, caused substantial transcriptional consequences within 2 hours, activating death pathways and cellular stress responses that fundamentally altered the infection context (Fig 7). Additionally, FSK partially mimicked the effects of MAP infection itself, maintaining expression of metabolic markers like *SHPK* and counteracting the spatial reorganization.

These observations corroborate previous reports of FSK-induced inflammatory responses mediated by IL-8 activation in colonic epithelial cells [80], but contrast with studies documenting anti-inflammatory effects of FSK treatment in neuronal and chondrocyte populations [81,82]. This contradiction is particularly notable given FSK’s role as an essential supplement for promoting crypt-villus morphogenesis in air-liquid interface organoid cultures [83], and its involvement in pathological cAMP-dependent secretory pathways resembling cholera toxin-induced diarrhea [84–86]. Given that the context-dependent nature of cAMP signaling across cellular environments creates confounding effects, we excluded FSK from our infection study, recommending alternative swelling strategies including osmotic manipulation or mechanical techniques.

Several limitations of our study warrant consideration. The single 48-hour timepoint prevents assessment of temporal dynamics, and our sample size, while adequate for detecting major transcriptional changes, may have limited power to detect subtle infection-specific genes given organoid variability. The transcriptomic approach cannot capture protein-level changes or metabolic flux, while bulk RNA-seq obscures cellular heterogeneity. Using one organoid line at passage number 13 and one MAP strain limits generalizability, and this reductionist model excludes immune cells, vasculature, and enteric neurons present in intestinal tissue.

Our findings have practical implications for organoid infection experiments. To minimize temporal confounding, studies should focus on (1) early infection processes when metabolic functions are more robust, (2) use time-course sampling instead of single timepoints, and (3) include multiple control groups. Given FSK’s substantial off-target effects, researchers should prioritize alternative microinjection approaches or include mandatory FSK-only controls. Gene set and pathway analyses demonstrate superior robustness to temporal confounding compared to individual gene approaches, with spatial gene set analysis offering promise for improving pathogenesis understanding in organoid systems.

Future research should prioritize advancing culture technologies including perfusion systems, optimized media formulations, and co-culture systems incorporating microbiota and immune cells. Comparative transcriptomic analyses across infection methods (microinjection, apical-out, monolayer) would help identify approaches that minimize technical artifacts. Investigating *SHPK’s* functional role in MAP infection and systematically screening the C3 gene set for alternative infection markers could identify robust biomarkers less susceptible to technical artifacts.

While our study reveals significant challenges inherent to organoid-based infection models, it provides a roadmap for optimization. By acknowledging and addressing temporal confounding, pharmacological artifacts, and culture-induced instability, the field can harness organoid advantages while minimizing limitations. The future of organoid infection research lies not in treating these models as perfect replicas of *in vivo* tissue, but rather in understanding their specific strengths and constraints, designing experiments within these boundaries, and integrating organoid data with complementary approaches to build comprehensive understanding of host-pathogen interactions.

## Conclusion

To our knowledge, this study pioneers the use of human intestinal organoids to model MAP infection and characterize epithelial transcriptomic responses, providing essential methodological foundations for investigating MAP’s potential role in Crohn’s disease pathogenesis. However, it also identifies fundamental challenges in organoid-based infection models that have important implications for the broader field of host-pathogen interaction research. Our transcriptomic analysis indicates that temporal transcriptional dynamics are the dominant signal in this system, which may obscure pathogen-specific responses. The progressive loss of mature intestinal functions, combined with activation of GC-rich genes potentially reflecting chromatin remodeling and spatial transcriptional reorganization, creates a complex and evolving cellular context that challenges interpretation of infection experiments at extended time points. FSK-mediated swelling, while technically convenient, introduces substantial confounding through activation of stress, apoptotic, and inflammatory pathways.

Although no single gene showed statistically robust differential expression, gene set-level analysis revealed that MAP infection counteracts temporal transcriptional drift, maintaining metabolic gene expression and enterocyte differentiation markers along the crypt-villus axis that otherwise decline during culture. This counter-regulatory response may suggest that MAP manipulates host cell metabolism to create a permissive intracellular niche. Our findings emphasize the critical importance of appropriate experimental controls, prioritizing early time points when metabolic functions remain robust, and skepticism toward pharmacological manipulation approaches that may introduce confounding effects. While this study exposes significant limitations of current organoid culture systems, it provides a framework for optimization and establishes realistic expectations for organoid-based research by acknowledging both the strengths and inherent constraints of the model.

## Supporting information

S1 Fig Sample characteristics. A) Mean gene expression levels across all 31 samples. B) Standard deviation of gene expression across samples, indicating variability within and between groups. C) Distribution of gene expression values across samples. Intermediate-expressed genes show progressive activation over time. n = 3 technical replicates per group except MAP48 (n = 4).
(DOCX)

S2 Fig Temporal correlation patterns of metagene expression across experimental conditions. The heatmap displays Pearson correlation coefficients between metagene expression patterns across all 31 samples, with values ranging from -1 (blue) to +1 (red). Hierarchical clustering was applied to both rows and columns. The analysis reveals distinct temporal clustering, with strong positive correlations (red) observed between samples from different experimental groups primarily at the 2 h time point. Samples from 2 h and the 24 h time point correlate negatively (blue) with the 48 h time point, while the 48 h time point shows consistent positive correlations. This temporal pattern is observed across all experimental conditions, indicating that the dynamics of gene expression changes are primarily driven by time rather than treatment. n = 3 technical replicates per group except MAP48 (n = 4); MAP-infected organoids were profiled at 48 h only.
(DOCX)

S3 Fig Zonation of the crypt-villus axis. **(A)** SOM analysis of expression data from Harnik et al. [44] identified six distinct gene clusters (AM-FM) through unsupervised clustering. Genes within each cluster exhibit similar expression patterns across the crypt-villus axis; representative genes are shown for each cluster. **(B)** Mean expression scores for cluster D and cluster E genes across crypts and six villus zones (error bars: standard deviation). **(C)** Differential expression analysis of gene sets between indicated sample regions across all clusters. FDR values were calculated using the Benjamini-Hochberg correction method; significant associations were identified at FDR < 0.05.
(DOCX)

S4 Fig MAP-specific and forskolin-responsive gene expression patterns. Differential expression analysis between MAP-infected samples at 48 h (MAP48, n = 4) and combined control samples (CON48 + PBS48, n = 6), performed using CAMERA (limma). Genes shown are significantly downregulated in control conditions (p < 0.001, uncorrected) but maintained expression in MAP-infected organoids. Two distinct gene clusters are highlighted: Cluster X contains genes upregulated by forskolin (FSK) treatment at early time points, while Cluster Y represents genes that maintain expression under FSK treatment at later time points and exhibit particularly strong MAP-specific regulatory effects. Notable genes include sedoheptulokinase (SHPK/CARKL), which shows the strongest MAP-specific regulation. The analysis demonstrates that forskolin treatment produces transcriptional effects that partially overlap with MAP infection responses.
(DOCX)

S5 Fig Comparison of MAP-infected and PBS-injected organoids at 48 hours. Differential expression of gene sets C1–C5 comparing MAP-infected vs. PBS-injected samples at 48 h (MAP48 vs. PBS48), assessed using CAMERA (limma). MAP infection induced upregulation across all crypt-villus zones relative to PBS controls, with statistical significance reached for crypt-proximal sets C1 and C2. FDR values were calculated using the Benjamini-Hochberg method; significant associations were identified at FDR < 0.05. n = 3 (PBS48); n = 4 (MAP48). Gene numbers may vary due to missing expression values in individual samples.
(DOCX)

S1 Data Raw RNA-seq count data. (ZIP)

S2 Data Gene lists of gene sets A-D. (ZIP)
