# Supplementary material for: Culture-specific transcriptional drifts limit the fidelity of organoid infection models
Source: PLoS Pathog. 2026 Jun 4;22(6):e1014321. doi: 10.1371/journal.ppat.1014321 (PMC13252844; doi:10.1371/journal.ppat.1014321)
Supplement: S1 Fig — A) Mean gene expression levels across all 31 samples. B) Standard deviation of gene expression across samples, indicating variability within and between groups. C) Distribution of gene expression values across samples. Intermediate-expressed genes show progressive activation over time. n = 3 technical replicates per group except MAP48 (n = 4). (DOCX) [file ppat.1014321.s001.docx]

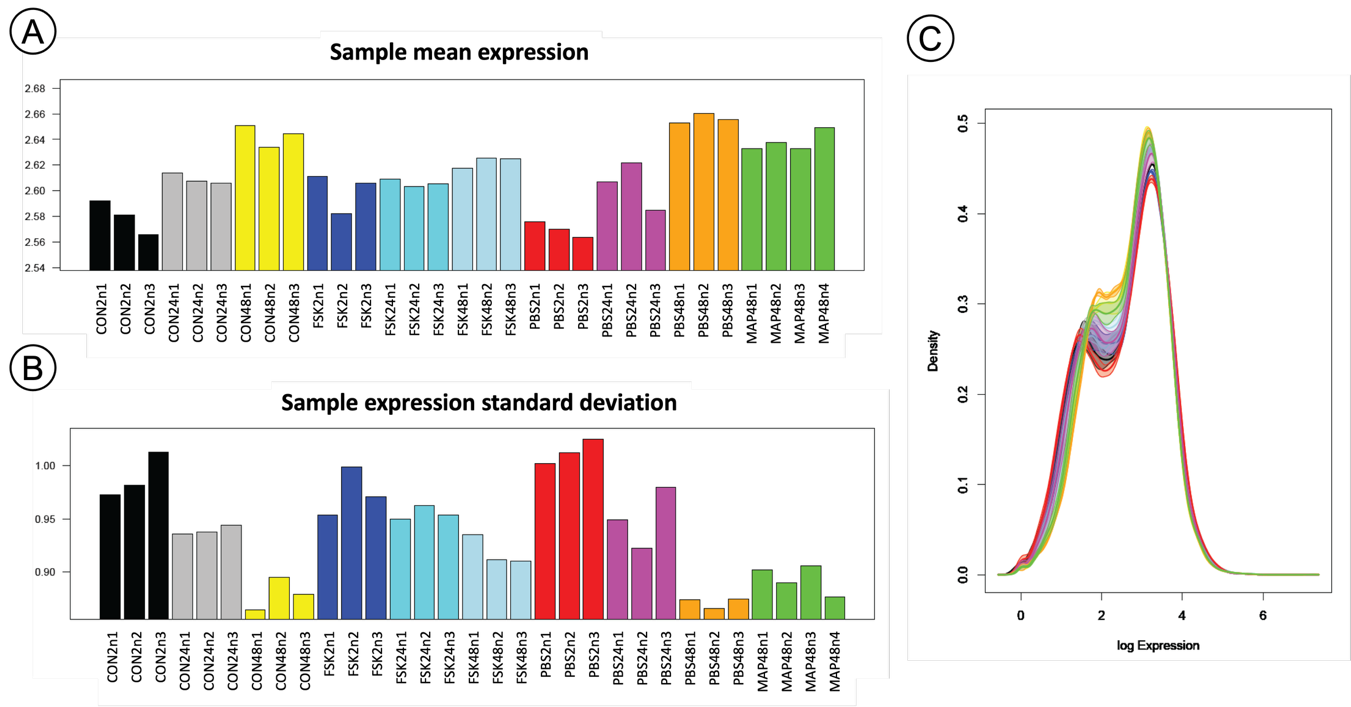


*Fig S1* **Sample characteristics.** A) Mean gene expression levels across all 31 samples. B) Standard deviation of gene expression across samples, indicating variability within and between groups. C) Distribution of gene expression values across samples. Intermediate-expressed genes show progressive activation over time. n=3 technical replicates per group except MAP48 (n=4).
