# Supplementary material for: Culture-specific transcriptional drifts limit the fidelity of organoid infection models
Source: PLoS Pathog. 2026 Jun 4;22(6):e1014321. doi: 10.1371/journal.ppat.1014321 (PMC13252844; doi:10.1371/journal.ppat.1014321)
Supplement: S2 Fig — The heatmap displays Pearson correlation coefficients between metagene expression patterns across all 31 samples, with values ranging from -1 (blue) to +1 (red). Hierarchical clustering was applied to both rows and columns. The analysis reveals distinct temporal clustering, with strong positive correlations (red) observed between samples from different experimental groups primarily at the 2 h time point. Samples from 2 h and the 24 h time point correlate negatively (blue) with the 48 h time point, while the 48 h time point shows consistent positive correlations. This temporal pattern is observed across all experimental conditions, indicating that the dynamics of gene expression changes are primarily driven by time rather than treatment. n = 3 technical replicates per group except MAP48 (n = 4); MAP-infected organoids were profiled at 48 h only. (DOCX) [file ppat.1014321.s002.docx]

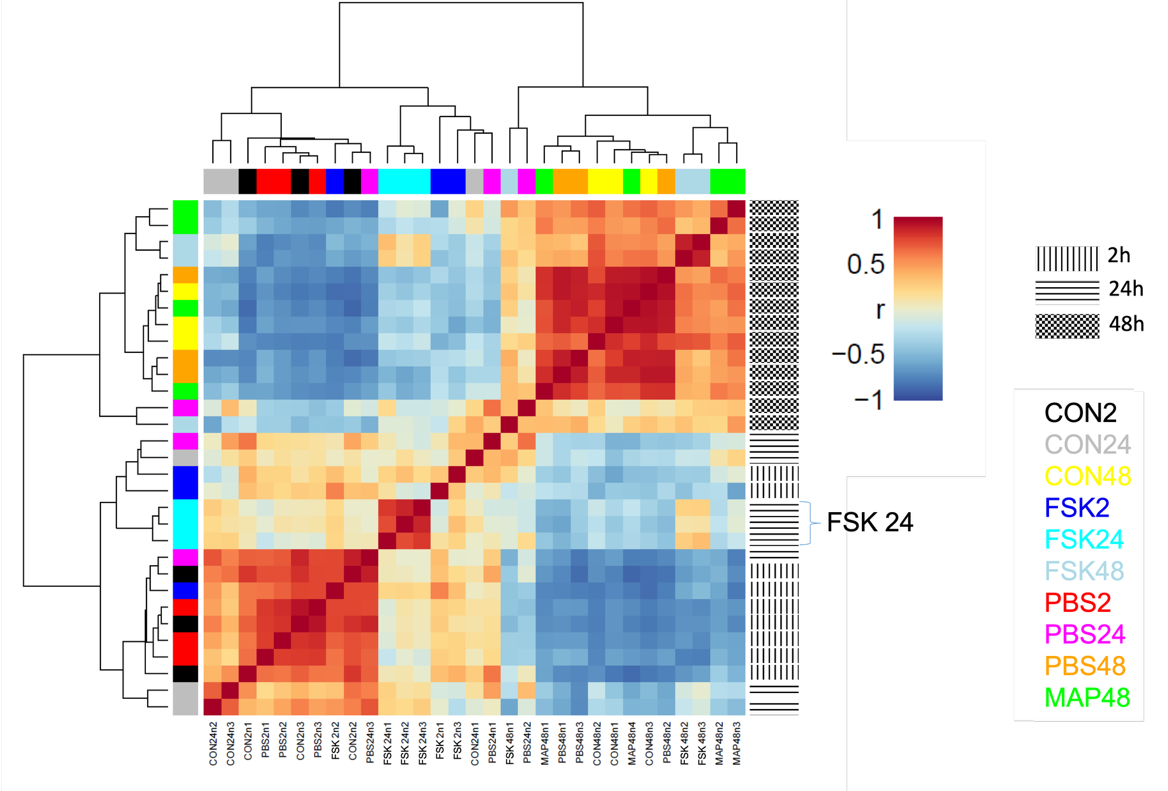


*Fig S2* **Temporal correlation patterns of metagene expression across experimental conditions.** The heatmap displays Pearson correlation coefficients between metagene expression patterns across all 31 samples, with values ranging from -1 (blue) to +1 (red). Hierarchical clustering was applied to both rows and columns. The analysis reveals distinct temporal clustering, with strong positive correlations (red) observed between samples from different experimental groups primarily at the 2 h time point. Samples from 2 h and the 24 h time point correlate negatively (blue) with the 48 h time point, while the 48 h time point shows consistent positive correlations. This temporal pattern is observed across all experimental conditions, indicating that the dynamics of gene expression changes are primarily driven by time rather than treatment. n=3 technical replicates per group except MAP48 (n=4); MAP-infected organoids were profiled at 48 h only.
