# Supplementary material for: Culture-specific transcriptional drifts limit the fidelity of organoid infection models
Source: PLoS Pathog. 2026 Jun 4;22(6):e1014321. doi: 10.1371/journal.ppat.1014321 (PMC13252844; doi:10.1371/journal.ppat.1014321)
Supplement: S3 Fig — (A) SOM analysis of expression data from Harnik et al. [44] identified six distinct gene clusters (AM-FM) through unsupervised clustering. Genes within each cluster exhibit similar expression patterns across the crypt-villus axis; representative genes are shown for each cluster. (B) Mean expression scores for cluster D and cluster E genes across crypts and six villus zones (error bars: standard deviation). (C) Differential expression analysis of gene sets between indicated sample regions across all clusters. FDR values were calculated using the Benjamini-Hochberg correction method; significant associations were identified at FDR < 0.05. (DOCX) [file ppat.1014321.s003.docx]

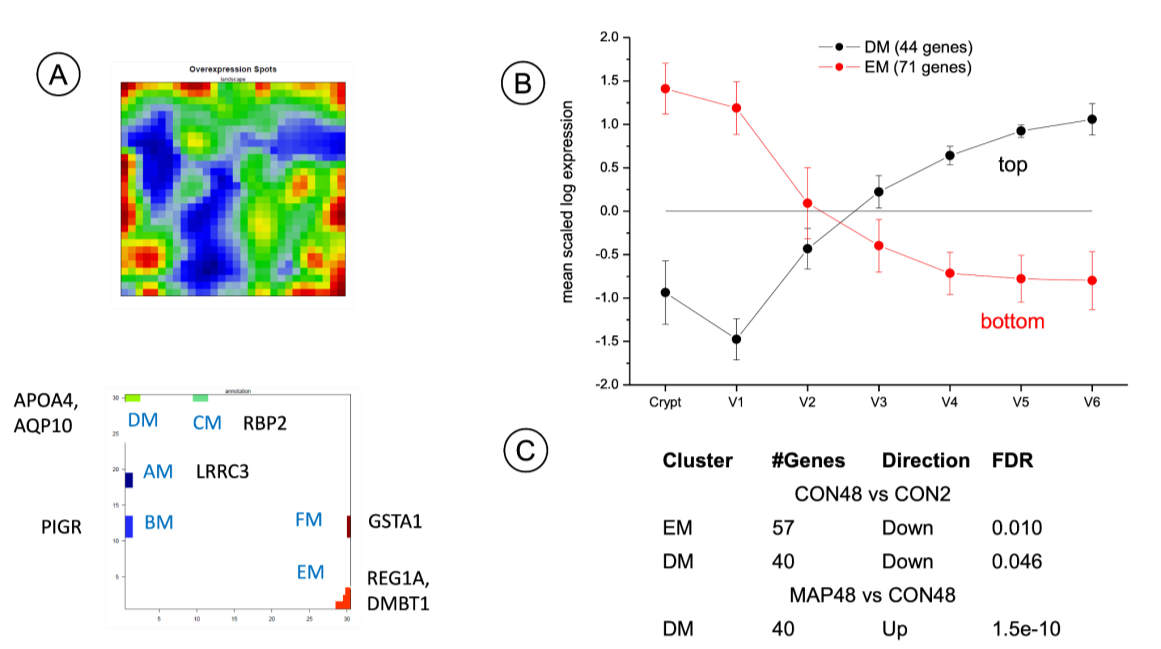


*Fig S3* **Zonation of the crypt-villus axis.** **(A)** SOM analysis of expression data from Harnik et al. [40] identified six distinct gene clusters (AM-FM) through unsupervised clustering. Genes within each cluster exhibit similar expression patterns across the crypt-villus axis; representative genes are shown for each cluster. **(B)** Mean expression scores for cluster D and cluster E genes across crypts and six villus zones (error bars: standard deviation). **(C)** Differential expression analysis of gene sets between indicated sample regions across all clusters. FDR values were calculated using the Benjamini-Hochberg correction method; significant associations were identified at FDR < 0.05.
