# Supplementary material for: Culture-specific transcriptional drifts limit the fidelity of organoid infection models
Source: PLoS Pathog. 2026 Jun 4;22(6):e1014321. doi: 10.1371/journal.ppat.1014321 (PMC13252844; doi:10.1371/journal.ppat.1014321)
Supplement: S4 Fig — Differential expression analysis between MAP-infected samples at 48 h (MAP48, n = 4) and combined control samples (CON48 + PBS48, n = 6), performed using CAMERA (limma). Genes shown are significantly downregulated in control conditions (p < 0.001, uncorrected) but maintained expression in MAP-infected organoids. Two distinct gene clusters are highlighted: Cluster X contains genes upregulated by forskolin (FSK) treatment at early time points, while Cluster Y represents genes that maintain expression under FSK treatment at later time points and exhibit particularly strong MAP-specific regulatory effects. Notable genes include sedoheptulokinase (SHPK/CARKL), which shows the strongest MAP-specific regulation. The analysis demonstrates that forskolin treatment produces transcriptional effects that partially overlap with MAP infection responses. (DOCX) [file ppat.1014321.s004.docx]

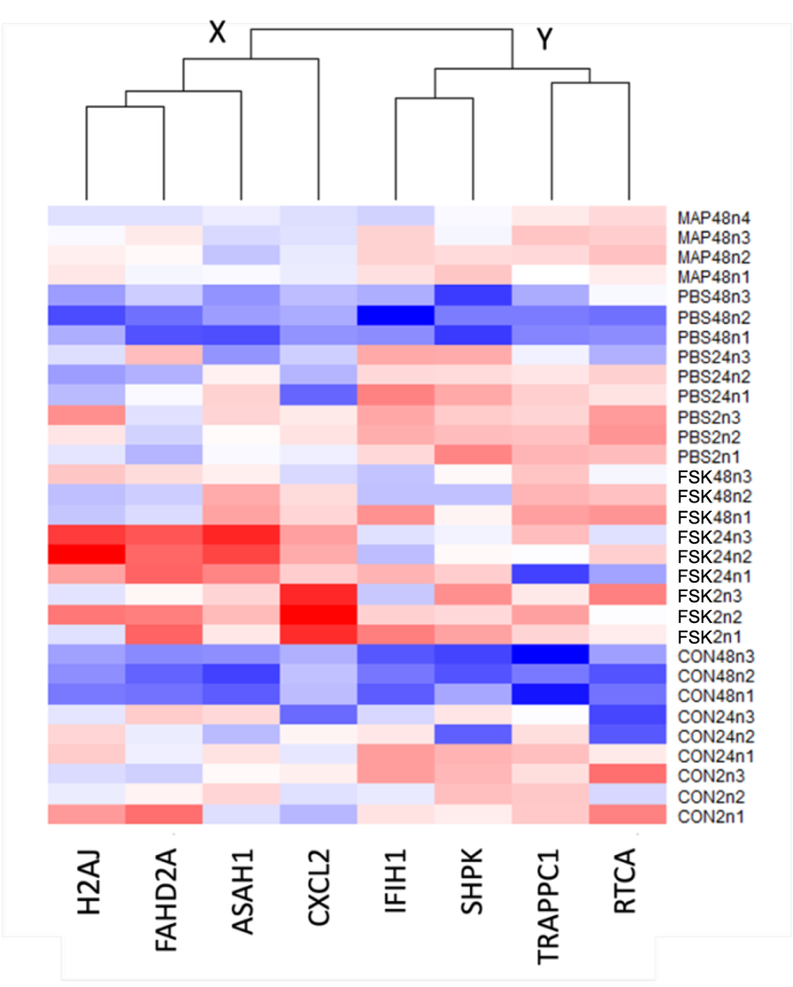


*Fig S4* **MAP-specific and forskolin-responsive gene expression patterns.** Differential expression analysis between MAP-infected samples at 48 h (MAP48, n=4) and combined control samples (CON48+PBS48, n=6). Genes shown are significantly downregulated in control conditions (p < 0.001, uncorrected) but maintained expression in MAP-infected organoids. Two distinct gene clusters are highlighted: Cluster X contains genes upregulated by forskolin (FSK) treatment at early time points, while Cluster Y represents genes that maintain expression under FSK treatment at later time points and exhibit particularly strong MAP-specific regulatory effects. Notable genes include sedoheptulokinase (SHPK/CARKL), which shows the strongest MAP-specific regulation. The analysis demonstrates that forskolin treatment produces transcriptional effects that partially overlap with MAP infection responses.
