# Supplementary material for: Culture-specific transcriptional drifts limit the fidelity of organoid infection models
Source: PLoS Pathog. 2026 Jun 4;22(6):e1014321. doi: 10.1371/journal.ppat.1014321 (PMC13252844; doi:10.1371/journal.ppat.1014321)
Supplement: S5 Fig — Differential expression of gene sets C1–C5 comparing MAP-infected vs. PBS-injected samples at 48 h (MAP48 vs. PBS48), assessed using CAMERA (limma). MAP infection induced upregulation across all crypt-villus zones relative to PBS controls, with statistical significance reached for crypt-proximal sets C1 and C2. FDR values were calculated using the Benjamini-Hochberg method; significant associations were identified at FDR < 0.05. n = 3 (PBS48); n = 4 (MAP48). Gene numbers may vary due to missing expression values in individual samples. (DOCX) [file ppat.1014321.s005.docx]

*
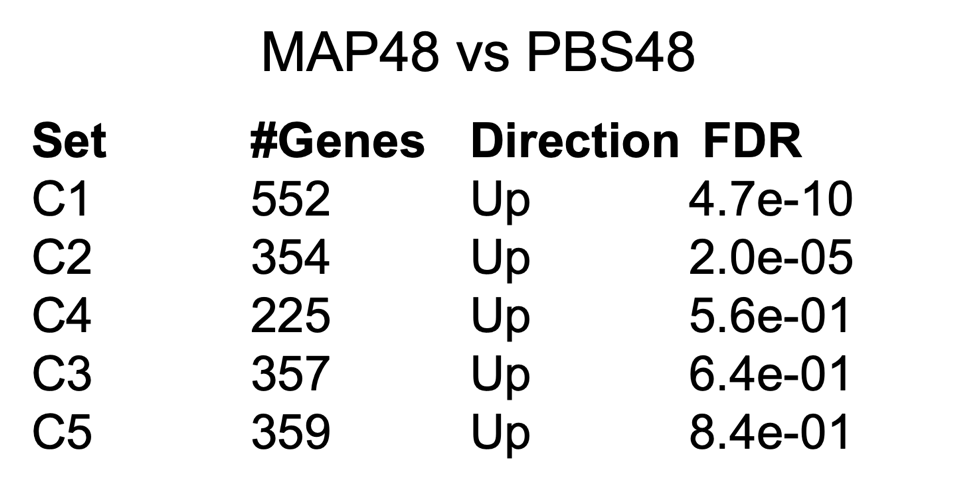
*

*Fig S5* **Comparison of MAP-infected and PBS-injected organoids at 48 hours.** Differential expression of gene sets C1–C5 comparing MAP-infected vs. PBS-injected samples at 48 h (MAP48 vs. PBS48), assessed using CAMERA (limma). MAP infection induced upregulation across all crypt-villus zones relative to PBS controls, with statistical significance reached for crypt-proximal sets C1 and C2. FDR values were calculated using the Benjamini-Hochberg method; significant associations were identified at FDR < 0.05. n=3 (PBS48); n=4 (MAP48). Gene numbers may vary due to missing expression values in individual samples.
